# Sodium ion channel alkaloid resistance does not vary with toxicity in aposematic *Dendrobates* poison frogs: An examination of correlated trait evolution

**DOI:** 10.1371/journal.pone.0194265

**Published:** 2018-03-13

**Authors:** Michael L. Yuan, Ian J. Wang

**Affiliations:** 1 Department of Environmental Science, Policy, and Management, College of Natural Resources, University of California, Berkeley, California, United States of America; 2 Museum of Vertebrate Zoology, University of California, Berkeley, California, United States of America; University of Sussex, UNITED KINGDOM

## Abstract

Spatial heterogeneity in the strength or agents of selection can lead to geographic variation in ecologically important phenotypes. Many dendrobatid frogs sequester alkaloid toxins from their diets and often exhibit fixed mutations at Na_V_1.4, a voltage-gated sodium ion channel associated with alkaloid toxin resistance. Yet previous studies have noted an absence of resistance mutations in individuals from several species known to sequester alkaloid toxins, suggesting possible intraspecific variation for alkaloid resistance in these species. Toxicity and alkaloid profiles vary substantially between populations in several poison frog species (genus *Dendrobates*) and are correlated with variation in a suite of related traits such as aposematic coloration. If resistance mutations are costly, due to alterations of channel gating properties, we expect that low toxicity populations will have reduced frequencies and potentially even the loss of resistance alleles. Here, we examine whether intraspecific variation in toxicity in three dendrobatid frogs is associated with intraspecific variation in alleles conferring toxin resistance. Specifically, we examine two species that display marked variation in toxicity throughout their native ranges (*Dendrobates pumilio* and *D*. *granuliferus*) and one species with reduced toxicity in its introduced range (*D*. *auratus*). However, we find no evidence for population-level variation in alkaloid resistance at Na_V_1.4. In fact, contrary to previous studies, we found that alkaloid resistance alleles were not absent in any populations of these species. All three species exhibit fixed alkaloid resistance mutations throughout their ranges, suggesting that these mutations are maintained even when alkaloid sequestration is substantially reduced.

## Introduction

Many species experience substantial variation in natural selection across their ranges leading to adaptive variation. In these scenarios, the relative costs and benefits of phenotypes under selection will vary across populations depending on local conditions, leading to local adaptation to optimize fitness tradeoffs [1,2]. Classically, variation in climate regimes often leads to widespread phenotypic variation tuned to local climate conditions (e.g. [3,4], and variation in predation pressure can drive the evolution of otherwise costly defenses in prey species (e.g. armor, chemical defense, etc.) [5–7] and resistance to defenses in predator species [8–10]. For example, predation pressure has driven the widespread evolution of chemical defenses [11–16]. However, resistance to chemical defenses is associated with strong fitness tradeoffs in both prey, which must sequester and maintain toxins, and predator species [10,15,17–19]. In both plants and sponges, increased chemical defenses are associated with decreased healing, growth, and recruitment rates [20–22], and in garter snakes, *Thamnophis sirtalis*, resistance to TTX-defense in prey species is associated with decreased motor performance [23,24]. Thus, widespread intraspecific variation in toxicity is observed in a variety of taxa, potentially resulting from variation in local ecological conditions (e.g. predation pressure or environmental toxin availability) [5,22,25,26]. Such variation in toxicity may lead directly to variation in the strength of selection maintaining toxin autoresistance across populations.

Adaptation to transmembrane protein-targeting toxins is highly costly and constrained due to the essential biological function of these protein families [17,27–31]. Thus, variation in toxin exposure often leads to variation in toxin resistance in response to fitness tradeoffs. A particularly well studied class of toxins are those which target the pore of voltage-gated sodium ion channels (Na_V_s)[17,32,33]. Populations of softshell clams, *Mya arenaria*, repeatedly exposed to saxitoxins (STX) have evolved resistance through a single Na_V_ amino acid replacement [34,35]. Similarly, populations of *T*. *sirtalis* that prey upon highly tetrodotoxin (TTX) defended *Taricha* newts have independently evolved resistance at Na_V_ paralogs [9,24,30]. In populations exposed to limited or no TTX, resistance is potentially deleterious due to reduced motor performance associated with TTX resistance [23,24]. Thus, population-level variation in resistance alleles throughout the range of *T*. *sirtalis* positively covaries with prey toxicity levels [5,9,23].

Poison frogs in the family Dendrobatidae have repeatedly evolved the capacity to sequester alkaloid toxins from their diets into specialized skin glands for chemical defense [16,36]. Three major classes of alkaloids found in poison frogs target Na_V_ channels: batrachotoxins (BTX), histrionicotoxins (HTX), and pumiliotoxins/allopumiliotoxins (PTX/aPTX)[16]. The utilization of dietary alkaloids as chemical defense requires the congruent evolution of resistance to toxins from prey and autoresistance to a species’ own toxins once sequestered [15,19,37]. Several lineages of poison frog have convergently evolved resistance to alkaloid toxins in at least one sodium ion channel, Na_V_1.4, in congruence with alkaloids as chemical defense [38]. Encoded by the *SCN4A* gene, Na_V_1.4 is one of six Na_V_ paralogs in amphibians [39]. Whereas several Na_V_ paralogs are only expressed in the central nervous system and thus protected from many alkaloid toxins that might otherwise interact with them by the blood-brain barrier, Na_V_1.4 is readily exposed to all sequestered toxins due to its expression in skeletal muscle tissues [30]. Previous work has identified two amino acid replacements, A446D and I433V –occurring in Domain I Subunit 6 (DI-S6), which forms part of the Na_V_1.4 pore region—associated with alkaloid resistance in *Dendrobates* [38]. Following Tarvin et al. [38], we refer to amino acids by their homologous position in the *Rattus norvegicus* Na_V_1.4 protein. Docking models have shown the Aspartic Acid 446 (446D) amino acid residue inhibits binding of alkaloid toxins (i.e. PTX and HTX), although evidence for Valine 433 (433V) is more equivocal [38] and resistance has not been experimentally tested for these mutations. The influence of these amino acid replacements on BTX resistance is minor [40], but BTX defense is not observed in *Dendrobates* and is limited to their sister genus, *Phyllobates* [16,38,41]. Additionally, amino acid changes in the S6 pore region of Na_V_ proteins may impose a fitness cost by substantially altering gating parameters, which can have consequences for normal channel function [42,43].

Despite widespread resistance mutations in other dendrobatids and both species being known to sequester alkaloid toxins [44], a recent study [38] reported an absence of alkaloid resistance in *Dendrobates* (= *Oophaga*) *pumilio* and *D*. (= *Adelphobates*) *galactonotus* Na_V_1.4 channels based on sequencing of single individuals in each species. If strong resistance to alkaloid toxins is costly to maintain, we should expect a pattern of covariation between alkaloid resistance alleles and toxicity, resulting in some populations having reduced Nav1.4 alkaloid resistance. Hence, this finding may reflect sampling drawn from populations with low alkaloid resistance allele frequencies rather than the absence of alkaloid resistance for the species entirely. Denser sampling of these and closely related species may reveal variation in alkaloid resistance traits across *Dendrobates*. Here, we examine whether the frequency of resistance alleles is reduced in lower toxicity populations relative to higher toxicity populations in three species of *Dendrobates*: *D*. *pumilio*, *D*. *granuliferus*, and *D*. *auratus*, focusing on two ecologically important substitutions (A446D and I433V) in the *SCN4A* gene that confer resistance to alkaloid toxins in other dendrobatids [38].

Several members of the *histrionicus* group, including *D*. *pumilio and D*. *granuliferus* (= *O*. *granulifera*), display dramatic variation in alkaloid toxicity throughout their ranges [26,45–47]. In both species, variation in both total toxicity and alkaloid profiles are strongly correlated with a suite of associated phenotypes, such as aposematic coloration [46,47], suggesting multi-trait selection driven by toxicity as a predation defense. *Dendrobates auratus* is a member of the *tinctorius* group and was intentionally introduced to Manoa, Hawaii from Isla de Taboga, Panama in 1932 [48]. The Manoa population displays substantially reduced alkaloid profiles relative to populations in the native range [25], allowing us to test whether selection against costly alkaloid resistance due to reduction of dietary alkaloids is associated with a loss of resistance alleles.

## Materials and methods

### Sample collection

We assembled a collection of whole livers from 35 *D*. *pumilio* individuals collected in 2006–2007 [49], 11 *D*. *granuliferus* collected in 2008 [46], and 15 *D*. *auratus* collected in 2007 and 2015. The *D*. *pumilio* samples represent six localities: Isla Bastimentos, Panama (N = 9); Isla Pastores, Panama (N = 4); Isla Colón, Panama (N = 7); Tortuguero, Costa Rica (N = 8); Puerto Viejo, Costa Rica (N = 5); and Guapiles, Costa Rica (N = 2). Toxicity in *Dendrobates* has been quantified through several methods, most notably mouse sensitivity assays in which alkaloid extracts are injected subcutaneously into lab mice (similar in principle to commonly used LD_50_ assays) [46,47] and gas chromatography with mass spectrometry (GC-MS) to characterize alkaloid profiles [26,50]. Subcutaneous mouse assays have been criticized because mice are not clearly analogous to the natural predators of these species and because predators receive toxins orally rather than subcutaneously in nature [51,52]. These assays also include the effects of alkaloids that do not interact with Na_V_1.4 channels. However, estimates from mouse assays generally correlate with the diversity and concentration of major alkaloids in these species [26,45–47]. Additionally, although not representative for the route of introduction in predators, for *Dendrobates* themselves subcutaneous injection is likely to expose toxins to more relevant tissues. Hereafter, we refer to mouse assays as ‘total toxicity’ and concentration of Na_V_1.4 acting alkaloids (i.e. PTX/aPTX and HTX) as ‘alkaloid profiles’. We discuss toxicity in the context of both datasets where literature is available for our sampled populations.

**Fig 1:**
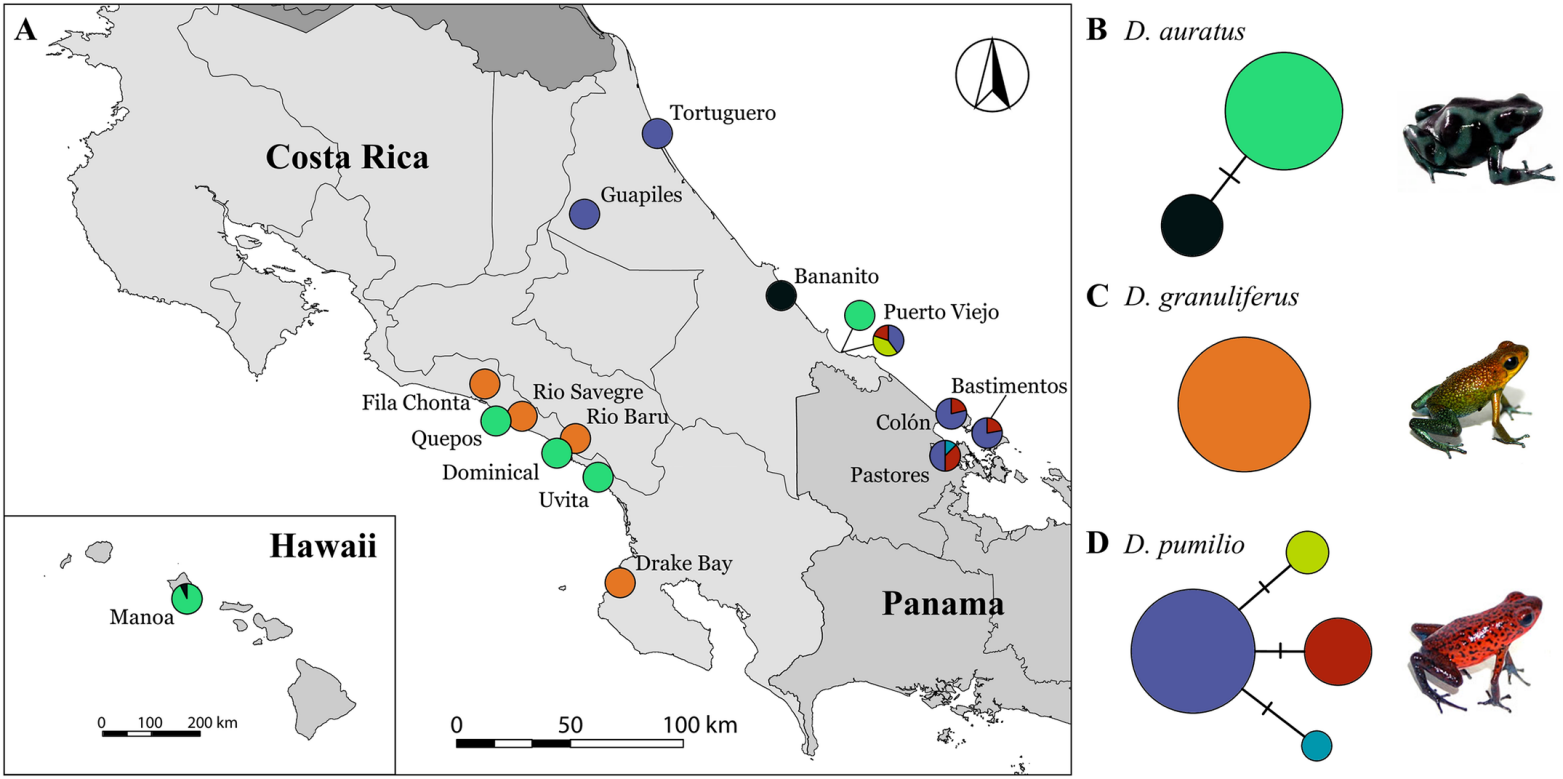
(A) Map of sampling localities. Relative haplotype frequencies for each sampling locality are denoted by pie charts. Colors for each haplotype correspond to haplotype networks displayed in B-D. (B-D) Haplotype networks for *SCN4A* in each species. Nodes sizes are scaled to represent the relative number of each haplotype and each unique haplotype is represented by a color. Photographs of each study species correspond to haplotype networks B-D respectively.

*Dendrobates pumilio* from the islands of Bocas del Toro, Panama include a wide variety of color morphs and toxicity levels [45,47,50,53]. Northwestern Isla Bastimentos represents one of the most toxic, whereas Isla Pastores and Isla Colón represent two of the least toxic populations in the region for both total toxicity and alkaloid profiles [45,47]. In fact, frogs on Isla Bastimentos are four to five times as toxic as frogs from Isla Colón in mouse assays [47]. The *D*. *granuliferus* samples represent four localities: Fila Chonta, Costa Rica (N = 2); Rio Savegre, Costa Rica (N = 1); Rio Baru, Costa Rica (N = 5); and Drake Bay, Costa Rica (N = 3). These localities represent the full range of color morphologies and associated total toxicity levels throughout the range of *D*. *granuliferus* [46] (Table 1). The Fila Chonta population is roughly 10% more toxic than the Rio Savegre population and about 60% more toxic than the Rio Baru and Drake Bay populations. Finally, we included tissues from *D*. *auratus* from five native localities and one introduced locality: Bananito, Costa Rica (N = 1); Puerto Viejo, Costa Rica (N = 2); Dominical, Costa Rica (N = 2); Uvita, Costa Rica (N = 2); Quepos, Costa Rica (N = 1); and Manoa, Hawai’i, USA (N = 7; Fig 1). We include sampling covering the Pacific (Quepos, Dominical, and Uvita) and Caribbean coasts (Bananito and Puerto Viejo) of Central America, which have different toxin profiles, and the less toxic introduced Manoa populations, which have reduced PTX/aPTX and lack HTX entirely [25].

**Table 1:** Total toxicity assays from Wang 2011 [46] for *D*. *granuliferus* sequenced in this study.

Sample	Locality	Mean	Standard Deviation
IW926	Fila Chonta	56.13	15.01
IW928	Fila Chonta	65.20	12.60
IW939	Rio Savegre	52.43	16.64
IW1005	Rio Baru	42.96	14.88
IW1006	Rio Baru	27.38	13.79
IW1007	Rio Baru	36.93	14.13
IW1008	Rio Baru	38.15	12.41
IW1012	Drake Bay	33.66	10.85
IW1013	Drake Bay	26.78	11.14
IW1014	Drake Bay	28.92	4.77

Toxicity data is represented as time to recovery (min) after subcutaneous injection of skin alkaloid extracts in a set of five lab mice. Individuals sequenced in this study represent a range of toxicities. These data were previously generated by Wang 2011 [46]. Data for IW1010 was not available.

### Nuclear sequencing

We extracted whole genomic DNA from ethanol-preserved liver samples using a DNeasy Blood and Tissue Kit (Qiagen) following the manufacturer’s protocol. We PCR-amplified the Domain I Subunit 6 (DI-S6) of the Na_V_1.4 sodium ion channel encoded by the *SCN4A* gene. We targeted this locus using primers +869F (5’-CTGCAGGYAAAACCTACATGG-3’;) and +1032R (5’-GATGTTTCTTTAGCTGTTCC-3’) [38]. For all samples, we conducted 25μL reactions using 2% dimethyl sulfoxide, 1X standard *Taq* reaction buffer (NEB), 1.5mM MgCl_2_, 0.2mM dNTPs, 0.2μM final concentrations for each primer, and 0.625U *Taq* polymerase (NEB). We ran PCRs at an initial temperature of 94°C for 2min, followed by 30 cycles of 94°C for 30s, 51°C for 30s, and 72°C for 30s, followed by a final extension at 72°C for 5min. We then purified amplicons using ExoSAP-IT (USB). Next, we performed cycle sequencing reactions using Big Dye v3.1 sequencing chemistry (Applied Biosystems) and sequenced Sephadex^™^ G-50 cleaned products on an ABI Automated 3730xl DNA Analyzer (Applied Biosystems). We examined electropherograms by eye and assembled sequences in Sequencher v4.8. Finally, we deposited all newly generated sequences in EMBL-ENA (accession numbers: LT984926-LT984986).

### Na_V_1.4 sequence analyses

We downloaded all available dendrobatid sequence data for Na_V_1.4 DI-S6 from Genbank (S1 Table). Additionally, we downloaded sequences from four non-dendrobatid hyloid frogs to serve as outgroups: *Espadarana callistomma*, *Lithodytes lineatus*, *Bufo nubulifer*, and *Gastrotheca litonedis*. We aligned previously available sequences with those generated in this study by amino acid sequence using MUSCLE [54]. To examine the evolutionary history of the Na_V_1.4 DI-S6 protein, we reconstructed a gene tree in BEAST2 [55]. We determined the best fitting partitioning scheme and associated models of nucleotide substitution using PARTITION FINDER 2 [56]. Specifically, we applied a K80+I model [57] to all codon positions. In BEAST, we ran two MCMC chains for 10,000,000 generations, sampling every 1,000 generations, and discarding 10,000 samples as burn-in. We assessed convergence of likelihood traces visually and by effective sample sizes (ESS) in Tracer v1.6.0 [58].

To examine *SCN4A* haplotype diversity across populations, we resolved haplotypes for heterozygous individuals with the PHASE algorithm in DNASP v5 [59]. We constructed median-joining networks [60] from phased haplotypes using POPART [61].

### Ancestral state reconstruction

We reconstructed the mtDNA phylogeny of Dendrobatidae using previously published 12S, 16S, ND1, and ND2 sequences. We used the same sequences as Tarvin et al. [38], with the addition of previously published mtDNA sequences for *D*. *auratus* (HQ290980) and *D*. *granuliferus* (DQ502035) not included in that study. In BEAST, we applied a GTR+I+Γ model to all genes and ran two MCMC chains for 10,000,000 generations, sampling every 1,000 generations and discarding 10,000 samples as burn-in. As with our *SCN4A* gene tree, we assessed convergence in Tracer v1.6.0 [58]. Using the mtDNA tree, we estimated ancestral states for alkaloid resistance associated amino acid replacements I433V and A446D [38]. We compared models of character evolution using the Akaike information criterion (AIC). We applied the Mk1 model (Markov k-state 1 parameter, in which a single rate is applied to all transitions), a Symmetrical Rate model (forward and reverse transitions have equal rates), and an All-Rates-Different model and estimated marginal likelihoods in the *ape* package in R v3.3.3 [62]. Because the All-Rates-Different and symmetrical rate models are identical for binary characters, we tested only two models for I433V: Mk1 and the All-Rates-Different/Symmetrical Rate model.

## Results

All three *Dendrobates* species exhibited little to no intraspecific sequence variation in *SCN4A* across their ranges (Fig 1). We recovered no non-synonymous variation in any of the three species. All sequences of *D*. *granuliferus* were identical. All Bocas del Toro island populations of *D*. *pumilio* (Isla Bastimentos—high total toxicity, Isla Pastores—low total toxicity, Isla Colón—low total toxicity) share *SCN4A* haplotypes with each other and with mainland Costa Rican populations. The introduced Manoa population of *D*. *auratus* shares haplotypes with mainland Costa Rican populations in its native range. All three species were fixed for the A446D amino acid replacement, and *D*. *auratus* was also fixed for the I433V amino acid replacement.

**Fig 2:**
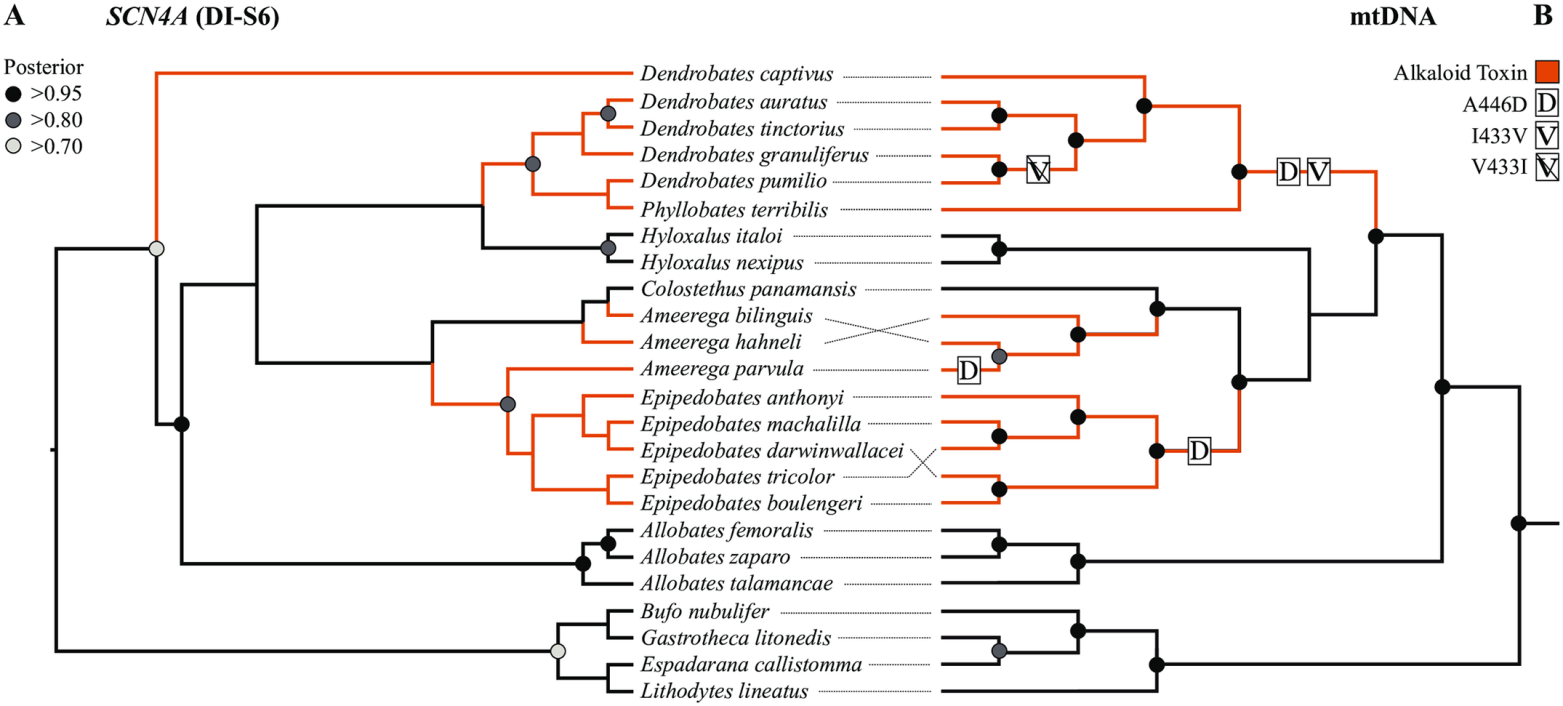
(A) Gene tree for Na_V_1.4 (*SCN4A*) DI-S6 inferred by BEAST. Branch lengths are transformed for ease of viewing and do not represent actual values. (B) mtDNA (12S, 16S, ND1, ND2) tree for Dendrobatidae. Nodes with posterior probability values above 0.70, 0.80, and 0.95 are denoted by shaded circles. Origins of resistance associated amino acid replacements, A446D and I433V, are denoted by boxes of D and V, respectively. Subsequent reversals to the ancestral state are denoted by strikethroughs. Lineages with alkaloid toxin defense are colored orange on both trees.

Six dendrobatid sequences downloaded from Genbank (presented by ref. [38]) were identical despite being sequenced from species in different genera: *Aromobates saltuensis* (KT989176.1), *D*. *galactonotus* (KT989187.1), *D*. *pumilio* (KT989188.1), *Phyllobates aurotaenia* (KT989190.1), *Colostethus fugax* (KT989192.1), and *Silverstoneia flotator* (KT989194.1). Given the highly unlikely nature of six otherwise distantly related species sharing entirely identical sequences, particularly synonymous sites, we excluded these samples from our analyses. Our mtDNA tree was well supported and congruent with previous trees built from those data (Fig 2) [38,44]. The *SCN4A* gene tree was relatively poorly supported, with the posterior probabilities of over half the nodes less than 0.70. However, we still recovered a relatively similar topology to our mtDNA tree and previously published mtDNA trees (Fig 2) [38,44]. Although the internal relationships within each clade differ, the relationships between the *Dendrobates*-*Phyllobates*, *Epipedobates*, *Hyloxalus*, and *Allobates* clades were congruent between the mtDNA and *SCN4A* gene trees. For *SCN4A*, *Ameerega parvula* is sister to *Epipedobates*, with which it shares the A446D replacement, rather than the non-resistant members of the *Ameerega-Colostethus* clade (Fig 2). Additionally, *D*. (= *Excidobates*) *captivus* did not cluster with the *Dendrobates-Phyllobates* clade but rather diverges at the base of the gene tree (Fig 2).

**Fig 3:**
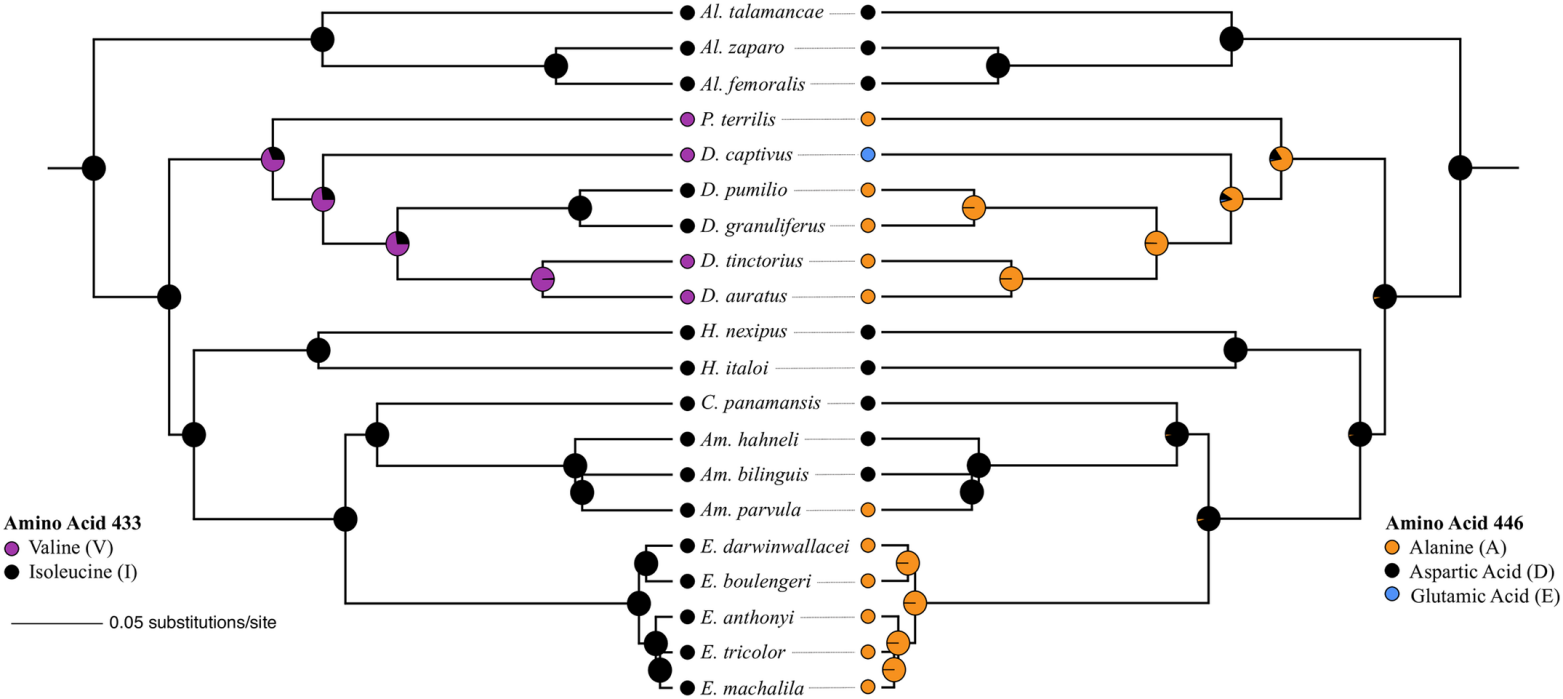
Ancestral state reconstructions for alkaloid resistance-conferring amino acid replacements. The underlying phylogeny was generated in BEAST using previously published 12S, 16S, ND1, and ND2 mtDNA sequences. Pie charts on nodes represent marginal likelihoods for ancestral character states.

The Mk1, single transition rate, model was the best performing ancestral state reconstruction model for both amino acids. We recovered a single origin for both the I433V and A446D amino acid replacements in the common ancestor of *Dendrobates* and *Phyllobates* (Fig 3). We also recovered a subsequent reversal of I433V in the *histrionicus* group and a secondary D446E replacement in *D*. *captivus* (Fig 3).

## Discussion

We found that variation in Na_V_1.4 alkaloid resistance at the sequence level is not associated with intraspecific variation in toxicity for three species of poison frogs that exhibit dramatic variation in toxicity across their ranges. There is no loss of resistance associated alleles in Hawaiian *D*. *auratus* despite having been introduced to Manoa 85 years ago [48], having reduced diversity and concentrations of HTX and PTX/aPTX [25], and having short generation times [63,64]. Although, the Manoa population was founded by the Isla de Taboga population off the Pacific coast of central Panama [48], several Manoa *SCN4A* sequences are identical to individuals collected from mainland Costa Rica (Fig 1). This suggests that little to no structure in *SCN4A* exists across large portions of the *D*. *auratus* range in Costa Rica and Panama. Similarly, all *SCN4A* sequences from *D*. *granuliferus* were identical, despite individuals from populations in the southern part of the range (Rio Savegre and Fila Chonta) being significantly less toxic than individuals from northern populations (Rio Baru and Drake Bay; Table 1) in both toxicity assays and alkaloid composition [46]. These southern populations completely lack several toxins found in the north, including two pumiliotoxins, one histrionicotoxin, and one indolizidine alkaloid [46]. Thus, neither overall alkaloid toxin profile nor total toxicity is associated with genetic variation at *SCN4A* in *D*. *granuliferus*. Although we recover some synonymous site variation in *D*. *pumilio*, this variation is not associated with toxicity or geographic structure (Fig 1), and our sequences revealed no non-synonymous site variation across all populations. Both the highly toxic Isla Bastimentos and the substantially less toxic Isla Pastores and Isla Colón populations share *SNC4A* haplotypes with each other and with mainland populations. Because we do not have individual level toxicity data for *D*. *pumilio* and *D*. *auratus*, the possibility exists that the individuals sampled here are not fully representative of the average toxicity in their respective populations because toxicity has been shown to vary between individuals, sexes, and temporally [25,26,50]. However, intrapopulation variation in toxicity and alkaloid profile is substantially less than interpopulation variation [25,26,47,50], suggesting that comparisons between populations should be robust to individual-level differences. Thus, in all three species, our data show that reduced toxicity does not lead to the loss of potentially costly alkaloid resistance mutations.

Our data do not support our initial hypothesis that the previously reported absence of the A446D resistance mutation in *D*. *pumilio* Na_V_1.4 [38] can be explained by intraspecific variation in toxicity. On the contrary, we find no evidence to support the conclusion of previous studies [38] that some dendrobatid frogs lack the A446D replacement even though they sequester toxic alkaloids. All three of our study species were fixed for the A446D amino acid replacement, including *D*. *pumilio* and closely related *D*. *granuliferus*. Although we did not sample *D*. *pumilio* in Nicaragua, our Tortuguero locality in northeastern Costa Rica is <35 km from the Nicaraguan border. Additionally, the previously reported *D*. *pumilio* Na_V_1.4 DI-S6 sequence (KT989188), as well as those for *D*. *galactinotus* (KT989187) and *Phyllobates aurotaenia* (KT989190), are among the six identical sequences discarded from our analyses. These discarded sequences show a phylogenetic placement outside of known toxin sequestering clades (S1 Fig), suggesting they were not derived from *Dendrobates* or *Phyllobates*. Our data supports the hypothesis that all members of *Dendrobates* share a single ancestral origin of the A446D mutation (Fig 3) rather than the multiple origins previously posited [38]. Therefore, in *D*. *captivus*, the Glutamic Acid 446 residue likely arose from a secondary mutation from Aspartic Acid (D446E) rather than from the previously suggested Alanine ancestral state [38]. Furthermore, given the concerns about the previously reported Na_V_1.4 DI-S6 sequence for *P*. *aurotaenia*, the single A446D origin may extend to a single origin for both *Dendrobates* and its sister group, *Phyllobates* (Fig 3). However, we did not resequence members of *Phyllobates* in this study.

Additionally, our results suggest that *D*. *auratus* is fixed for the I433V amino acid replacement. The I443V replacement has also been reported in the closely-related *D*. *tinctorius* as well as the more distantly related *D*. *captivus* and *P*. *terribilis*. Our data support a single origin of I433V followed by a reversal in the *histrionicus* group (Fig 3). However, we cannot definitively rule out multiple origins of the I433V replacement. Alkaloid profiles indicate that *D*. *auratus* is likely to be more toxic than *D*. *pumilio* and *D*. *granuliferus* [41]. It is possible that the additional replacement in *SNC4A* from I433V confers additional resistance that allows for greater toxicity in species such as *D*. *auratus* and *P*. *terribilis*, the most toxic known dendrobatid [41,65]. However, the toxicity of *D*. *captivus* has not been characterized, and support for I433V contributing to PTX and HTX resistance is equivocal [38]. A446D and I433V provide some additional resistance to BTX when experimentally co-expressed in mammalian cells with additional replacements unique to *P*. *terribilis*, although their influence is minor [40].

If alkaloid resistance is costly to maintain in dendrobatid frogs, why do low toxicity populations maintain resistance phenotypes? One possible explanation is that sodium-ion channel (Na_V_1.4) alkaloid resistance is not actually costly to maintain. Although I433V and A446D shift gating properties in Na_V_1.4 when expressed in mammalian cells, whether this shift has functional consequences in *Dendrobates* is unknown [40]. Still, these mutations occur in the otherwise highly functionally conserved pore region of Nav1.4, and toxin resistance is associated with fitness costs in other vertebrates [17,28,66]. For example, TTX resistance alleles are associated with decreased motor performance in *T*. *sirtalis* [24]. However, experimental evidence has demonstrated that even adaptive phenotypes that are usually associated with fitness trade-offs can sometimes arise through underlying mechanisms with no associated cost [67]. Alternatively, the benefits of alkaloid resistance may outweigh the fitness costs even at low toxin concentrations, thus necessitating its maintenance in low toxicity populations. However, alkaloid resistance is maintained even in the Isla Pastores and Isla Colón populations of *D*. *pumilio*, which appears to be no more toxic in toxicity assays than non-toxic *Allobates talamancae* controls in mouse assays [47]. Costly alkaloid resistance genotypes may be maintained to protect against alkaloid toxins in prey items even when toxic prey are minor dietary components or when toxins are not actively sequestered by *Dendrobates* populations. Studies of prey communities, their alkaloid toxin profiles, and prey selection by *Dendrobates* are necessary to assess this hypothesis. Finally, the maintenance of alkaloid resistance at Na_V_1.4 may be a phylogenetic artifact [68]. If the A446D replacement was fixed in the ancestor of modern dendrobatines (*Dendrobates* and *Phyllobates*), reversal would require a *de novo* mutation, a stochastic event that simply may not have occurred in our study species. This scenario may explain the maintenance of the A446D replacement even if it is associated with a fitness tradeoff. Future studies on the fitness consequences of these mutations in various environmental contexts are needed to differentiate between these scenarios.

## Conclusions

Contrary to expectations, we found no evidence for geographic variation in alkaloid resistance in the Na_V_1.4 voltage-gated sodium channel in *D*. *pumilio*, *D*. *granuliferus*, or *D*. *auratus*, even though each species displays dramatic variation in toxicity. Rather, our data suggest that these mutations are maintained even in populations where skin alkaloid profiles are substantially reduced, indicating either a lack of substantial fitness tradeoffs or the lack of reversal mutations. Further work is needed to determine the relative fitness costs, if any, for maintaining high level resistance despite low level toxicity. Additionally, we found no evidence for the previously reported absence of alkaloid resistance alleles in *Dendrobates* frogs [38]. Instead, our data support a single origin of the A446D amino acid replacement for the *Dendrobates-Phyllobates* clade and a single origin followed by a reversal for the I433V replacement. This single origin of initial alkaloid resistance is coincident with the hypothesized origin of alkaloid sequestration in the common ancestor of *Dendrobates* and *Phyllobates* frogs.

## Supporting information

S1 Fig Gene tree for Na_V_1.4 (*SCN4A*) DI-S6 inferred by BEAST including discarded sequences from Genbank. All discarded sequences (shown in red) are identical in sequence and do not fall within known toxic clades of Dendrobatids. Posteriors above 0.80 are denoted.
(PDF)

S1 Table Genbank accession numbers for previously published Na_V_1.4 DI-S6 and mtDNA (12S, 16S, ND1, and ND2) sequences used in this study. (DOCX)
