# Supplementary figures and images for: Sodium ion channel alkaloid resistance does not vary with toxicity in aposematic Dendrobates poison frogs: An examination of correlated trait evolution
Source: PLoS One. 2018 Mar 13;13(3):e0194265. doi: 10.1371/journal.pone.0194265 (PMC5849323; doi:10.1371/journal.pone.0194265)

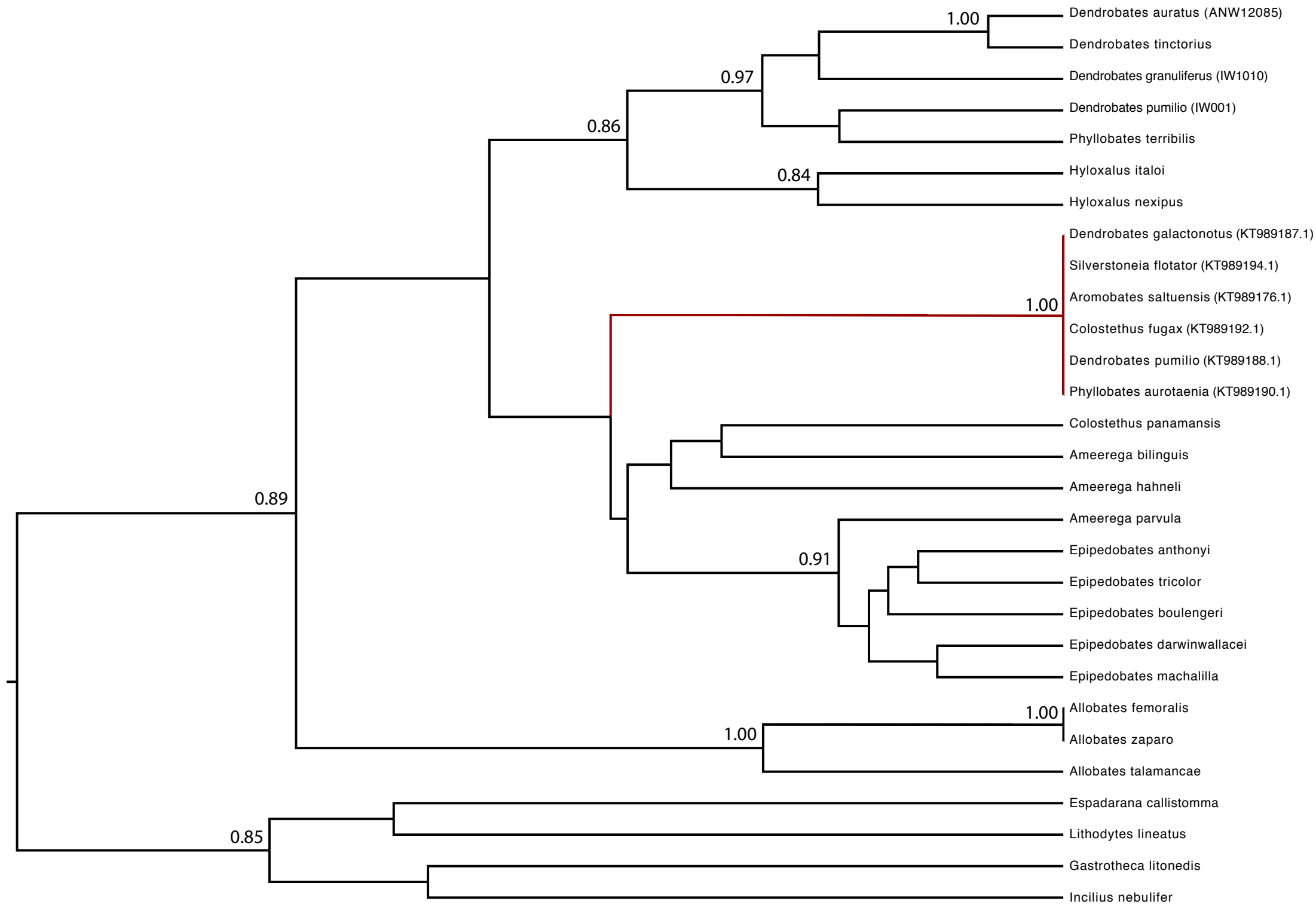

0.0060 substitutions/site

Supplement: S1 Fig — All discarded sequences (shown in red) are identical in sequence and do not fall within known toxic clades of Dendrobatids. Posteriors above 0.80 are denoted. (PDF) [file pone.0194265.s001.pdf]
