# Supplementary material for: Sodium ion channel alkaloid resistance does not vary with toxicity in aposematic Dendrobates poison frogs: An examination of correlated trait evolution
Source: PLoS One. 2018 Mar 13;13(3):e0194265. doi: 10.1371/journal.pone.0194265 (PMC5849323; doi:10.1371/journal.pone.0194265)
Supplement: S1 Table — (DOCX) [file pone.0194265.s002.docx]

**S1 Table** Genbank accession numbers for Na_V_1.4 DI-S6 and mtDNA (12S, 16S, ND1, and ND2) sequences used in this study. Previously published sequences were derived from references [1–5].

| **Species** | **Nav1.4 DI-S6** | **mtDNA** |
| --- | --- | --- |
| *Allobates femoralis* | KT989177 | HQ290951 |
| *Allobates talamancae* | KT989178 | HQ290974 |
| *Allobates zaparo* | KT989179 | HQ291003 |
| *Ameerega bilinguis* | KT989180 | HQ290996 |
| *Ameerega hahneli* | KT989181 | HQ290998 |
| *Ameerega parvula* | KT989182 | HQ290999 |
| *Bufo (=Incilius) nubiuifer* | KT989172 | HQ290945 |
| *Colostethus panamansis* | KT989193 | HQ290968 |
| *Dendrobates (=Excidobates) captivus* | KT989186 | HQ290982 |
| *Dendrobates (=Oophaga) pumilio* | TBD | HQ290988 |
| *Dendrobates auratus* | TBD | HQ290980 |
| *Dendrobates granuliferus (=Oophaga granulifera)* | TBD | DQ502035 |
| *Dendrobates tinctorius* | KT989189 | HQ290991 |
| *Epipedobates anthonyi* | KT989195 | HQ290995 |
| *Epipedobates boulengeri* | KT989196 | HQ290997 |
| *Epipedobates darwinwallacei* | KT989197 | HQ291000 |
| *Epipedobates machalila* | KT989198 | HQ290964 |
| *Epipedobates tricolor* | KT989199 | HQ291001 |
| *Espadarana callistomma* | KT989175 | EU663340, EU663076, EU662981 |
| *Gastrotheca litonedis* | KT989173 | DQ679247, KJ489515 |
| *Hyloxalus italoi* | KT989184 | HQ290972 |
| *Hyloxalus nexipus* | KT989185 | HQ290965 |
| *Lithodytes lineatus* | KT989174 | HQ290949 |
| *Phyllobates terribilis* | KT989191 | HQ291006 |

**References**

1. Tarvin RD, Santos JC, O’Connell LA, Zakon HH, Cannatella DC. Convergent Substitutions in a Sodium Channel Suggest Multiple Origins of Toxin Resistance in Poison Frogs. Mol Biol Evol. 2016;33: 1068–1081. doi:10.1093/molbev/msv350

2. Wiens JJ, Kuczynski CA, Duellman WE, Reeder TW. Loss and re-evolution of complex life cycles in marsupial frogs: does ancestral trait reconstruction mislead? Evolution. 2007;61: 1886–1899. doi:10.1111/j.1558-5646.2007.00159.x

3. Grant T, Frost DR, Caldwell JP, Gagliardo R, Haddad CFB, Kok PJR, et al. Phylogenetic systematics of dart-poison frogs and their relatives (Amphibia, Athesphatanura, Dendrobatidae). Bulletin of the AMNH ; no. 299. Phylogenetics of dart-poison frogs. 2006; Available: http://digitallibrary.amnh.org/handle/2246/5803

4. Guayasamin JM, Castroviejo-Fisher S, Ayarzagüena J, Trueb L, Vilà C. Phylogenetic relationships of glassfrogs (Centrolenidae) based on mitochondrial and nuclear genes. Mol Phylogenet Evol. 2008;48: 574–595. doi:10.1016/j.ympev.2008.04.012

5. Santos JC, Cannatella DC. Phenotypic integration emerges from aposematism and scale in poison frogs. PNAS. 2011;108: 6175–6180. doi:10.1073/pnas.1010952108
